# Evolution of Complex Target SELEX to Identify Aptamers against Mammalian Cell-Surface Antigens

**DOI:** 10.3390/molecules22020215

**Published:** 2017-01-30

**Authors:** Prabodhika Mallikaratchy

**Affiliations:** 1 Department of Chemistry, Lehman College, The City University of New York, 250 Bedford Park Blvd. West, Bronx, NY 10468, USA; prabodhika.mallikaratchy@lehman.cuny.edu; 2 Ph.D. Programs in Chemistry and Biochemistry, CUNY Graduate Center 365 Fifth Avenue, New York, NY 10016, USA; 3 Ph.D. Program in Molecular, Cellular and Developmental Biology, CUNY Graduate Center 365 Fifth Avenue, New York, NY 10016, USA

**Keywords:** SELEX, nucleic acid aptamers, cell-surface markers

## Abstract

The demand has increased for sophisticated molecular tools with improved detection limits. Such molecules should be simple in structure, yet stable enough for clinical applications. Nucleic acid aptamers (NAAs) represent a class of molecules able to meet this demand. In particular, aptamers, a class of small nucleic acid ligands that are composed of single-stranded modified/unmodified RNA/DNA molecules, can be evolved from a complex library using Systematic Evolution of Ligands by EXponential enrichment (SELEX) against almost any molecule. Since its introduction in 1990, in stages, SELEX technology has itself undergone several modifications, improving selection and broadening the repertoire of targets. This review summarizes these milestones that have pushed the field forward, allowing researchers to generate aptamers that can potentially be applied as therapeutic and diagnostic agents.

## 1. Introduction

The elevated expression level of cell-surface proteins in response to disease is a key concept underlying the development of target-specific molecular probes [1,2]. Cell-surface proteins are primarily engaged in signal transduction and ligand binding. In the disease state, receptor molecules at the cell surface respond to altered biological activity by exhibiting different levels of expression [3]. It is this differential expression that allows researchers and clinicians to distinguish between diseased and normal cells. Detecting this variable expression of surface markers has been extensively used in many clinical applications as an avenue to diagnose and treat specific diseases [4,5,6]. From a fundamental point of view, molecular probes able to recognize such markers would be a key advantage, and, indeed, the demand for such sophisticated molecular tools is increasing.

In particular, nucleic acid aptamers (NAAs) represent a class of molecules able to meet this demand. NAAs are synthetic DNA or RNA molecules with the ability to recognize a specific molecule with high affinity and specificity [7,8]. The nature of the molecular recognition between the aptamer and its target is based on non-covalent interactions. Specifically, electrostatic and hydrophobic interactions, as well as shape complementarity, govern the aptamer’s recognition of its target [9]. In the context of clinical applications, NAAs are attractive for their unique pharmacokinetics properties and their easily modifiable synthetic nature, which has allowed the incorporation of a wide variety of functional groups at precise positions in aptamer molecules without compromising their affinity or specificity [10,11,12,13]. This property is especially important for aptamers used in sensors, as diagnostic agents, or as drug conjugates. For example, based on a study conducted by Watson et al., clearance of an aptamer against L-selectin could be modulated by simply modifying an aptamer with high-molecular-weight poly-(ethylene glycol) in vivo, utilizing animal models to reduce renal clearance and extend circulation time [14]. These properties are unique to aptamers, and they have been successfully utilized in designing new versatile molecules aimed at a multitude of applications [15,16,17,18,19]. Most importantly, recent advances in oligonucleotide-based therapeutics, such as antisense technology and microRNA therapeutics, have spurred a renewed demand for the development of novel aptamer-based targeting molecules [20,21,22]. Utilization of NAAs as carriers of siRNA or microRNA can eliminate tedious conjugation methods, thus allowing one molecule to serve as both the drug and targeting agent. In fact, both target recognition and therapeutic moiety can be combined in one step using a solid-state synthesis in an automated oligonucleotide synthesizer. A number of examples have shown the versatility of aptamers discovered against cell-surface markers as delivery agents for microRNA, and siRNA, both in vitro and in vivo [7,23,24,25,26].

The concept of in vitro evolution of nucleic acid molecules was introduced in 1990 from three independent groups. Robertson and Joyce introduced in vitro selection of more efficient catalytic RNA compared to wild-type Tetrahymena ribozyme [27]. Tuerk and Gold reported the isolation of high-affinity RNA ligands against T4 DNA polymerase and coined the term Systematic Evolution of Ligands by EXponential enrichment, or SELEX [28]. Finally, Ellignton and Soztak demonstrated that RNA ligands could be enriched from a pool of RNA molecules against organic dye molecules and defined these artificial RNA ligands as aptamers [29]. Thus, by combining the rules of combinatorial library screening with in vitro evolution, these studies demonstrated, for the first time, that nucleic acid ligands could be isolated against a wide range of molecules. Since these initial reports of SELEX, many independent reports have shown that aptamers can be evolved from a complex library using SELEX against almost any molecule, especially molecules that are not known as oligonucleotide-binding molecules [7,19,22,30]. Establishing the SELEX method in generating hundreds of aptamers for use in therapeutics or diagnostics was initially successful [8]. Nonetheless, only one aptamer has thus far been approved by the FDA for neovascular age-related macular degeneration (Macugen (pegaptanib)) [31,32]. Several aptamers are currently being evaluated in various clinical trials, ranging from phase 1 to 3 [33].

**Figure 1:**
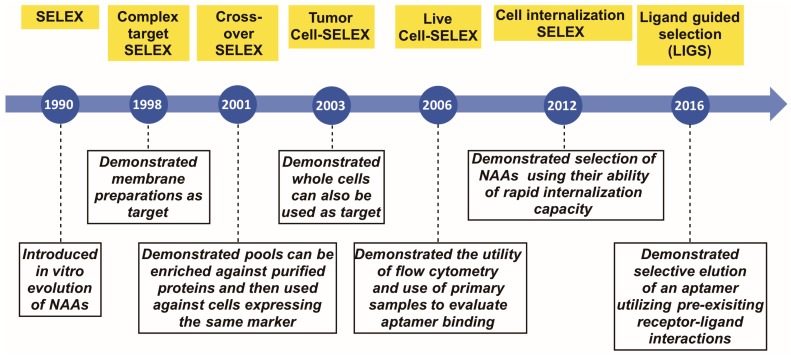
Progression of SELEX methods against complex targets.

The SELEX process involves three interconnected steps. Step one involves the repeated incubation of a library with a specific target to allow binding of high-affinity aptamers. Step two involves the separation of high- from low-affinity binders. Step three involves the amplification of high-affinity binders utilizing polymerase chain reaction (PCR). This process is repeated until the high-affinity aptamers are enriched in the selection pool. The basis of the SELEX process is mainly rooted in the ability of high-affinity binders to survive in a SELEX pool through several rounds of selection. External selection pressures to enhance selectivity and affinity of the survivors are implemented throughout the process. As discussed by Eaton et al., NAAs have been identified that can distinguish between l and d amino acids and between reduced and oxidized cofactors [9]. This demonstrates the level of specificity that can be achieved by aptamers. However, it is important to point out that such specificity can also be detrimental when a single purified protein is used as the SELEX target. SELEX is successful in generating NAA sequences towards a purified protein, but it is well known that the binding of these NAAs can only be mimicked in conditions used for aptamer selection. In other words, aptamers selected using purified protein may not recognize the same protein at its endogenous levels or conditions in a cell. Many aptamers were reported during the first decade after the introduction of SELEX, but most aptamers discovered against purified protein targets failed to recognize the same protein in its native environment. Nonetheless, a few aptamers have been successful and are worth mentioning. For example, an aptamer against extracellular domains of prostate-specific membrane antigen (PSMA) has been successful and used in many proof-of-concept applications [34,35,36]. As noted previously, the SELEX method itself has been updated many times by allowing modifications that changed or improved the selection process. This review will highlight advancements in SELEX technology towards complex targets (Figure 1), in particular those aptamers that may now potentially be applied as theranostic agents.

## 2. Complex Target SELEX

Successful selection of high-affinity aptamers depends on consistent, unperturbed presentation of the targeting epitope [37]. Therefore, during the SELEX process, it is necessary to maintain a uniform, folded state of the desired protein to successfully obtain high-affinity aptamers. Morris et al. identified this problem and developed a variant of the original SELEX called complex target SELEX, which particularly addresses the target [38]. For the first time, these authors demonstrated that human red blood cell membrane preparations (RBC ghosts) can be used in place of purified membrane proteins as long as the potential target(s) on RBC remained unchanged during the in vitro selection process [38]. Based on these results, it was postulated that the epitope remained unchanged, either by mis-folding or other unknown salt effects, thereby facilitating selective identification of aptamers against RBC membranes [38]. A similar approach was utilized for the original SELEX with 25 rounds using nitrocellulose filter binding as the method of separating high- from low-affinity binders. The approach carried out by Morris et al. led to the discovery of 41 sequences, which could be categorized into six distinct families based on common motifs among the sequences [38]. The truncated motifs modified with photo-crosslinking functional groups, followed by SDS-PAGE, and distinctly identified protein complexes specifically cross-linked to aptamers identified by complex target SELEX. This was the first study to demonstrate that (1) aptamers against multiple targets could be identified utilizing multi-epitope membrane preparations; (2) the SELEX library is sufficiently diverse to allow competition between potential aptamer ligands towards one target; (3) multiple aptamers could be selected towards multiple targets. The authors reported that the aptamers selected by complex target SELEX bind to proteins based on gel-electrophoresis analysis, but the authors did not identify the protein.

## 3. Crossover- or (Hybrid)-SELEX

To enhance the selection efficiency and avoid generating aptamers against potential co-receptors of the biomarker of interest was introduced through a variant of SELEX termed hybrid- or crossover-SELEX [39]. This approach is especially useful in generating NAAs against known biomarkers. Demonstrated by multiple groups, crossover-SELEX first enriches an aptamer pool against a whole cell expressing the target of interest, followed by further enrichment of the aptamers against the recombinant purified biomarker [40]. Using this method, an RNA aptamer against Tenascin C (TN-C), a glycoprotein that, in humans, is encoded by the *TNC* gene and is over-expressed on U251 glioblastoma cells, was identified [39]. A reverse crossover-SELEX was later employed to identify anti-Transferrin aptamers [41].

## 4. Tumor Cell SELEX

Later, Daniels et al. demonstrated that aptamers could be generated against antigens presented by a monolayer of tumor cells [42]. To accomplish this, a glioblastoma cell line, U251, was used as the target for a SELEX library of single-stranded DNA (ssDNA). It was shown that whole cells could be utilized as targets in SELEX experiments. With the objective of a priori identification of biologically important targets and the potential development of aptamers with in vivo targeting ability, the authors further demonstrated that an ssDNA pool could be evolved with 21 iterative rounds against a monolayer of glioblastoma cells. One aptamer identified during the selection, termed GBI-10, represented 10% of the sequences among sequences obtained by sequenced SELEX pool. Next, GBI-10 was used as a capturing agent and affinity purification was utilized to identify the target of the aptamer. The identified target of GBI-10 is Tenascin C, which is a hexameric protein expressed extracellularly in the tumor matrix. However, since the selection was performed at 4 °C, the identified aptamers did not recognize the target at physiological conditions.

By utilizing mono-layered tumor cells as the target, tumor cell SELEX demonstrated that aptamers could be generated against distinct known epitopes expressed on U251 cells. This study also demonstrated the use of affinity pull-down assays, followed by mass spectrometry to identify the aptamer’s target. The key significance of this method is the demonstration that NAAs can be developed against surface markers in their native state, which is a particularly important feature, given that these trans-membrane proteins could face the possibility of mis-folding in a purified form.

These examples show the ability of SELEX to identify aptamers against cell-surface proteins utilizing whole cells, thereby opening the path toward developing more sophisticated selection approaches.

## 5. Live Cell-SELEX Utilizing Flow Cytometry for Biomarker Discovery

To develop aptamer-based molecular tools able to distinguish differences between healthy and diseased cells at the molecular level, Shangguan et al. developed a variant of complex target SELEX termed live cell–SELEX [43]. Here, a counter (negative)-selection using an unrelated cell line was applied to enhance selection pressure and remove aptamers present in the SELEX pool that bind to receptors present in both positive and negative cell lines. Using this approach, five aptamers were identified against cultured lymphoblastic leukemia cells. By utilizing two cell lines and incorporating the negative selection, the authors hypothesized that new aptamers could be identified to recognize surface markers uniquely expressed on the positive cell line. Upon selecting the target cell line, the choice of the negative cell line predefines the nature of the molecular differences between the cell lines; thus, the aptamers will be evolved according to the predefined difference between the two cell lines [44]. This reasoning presupposes that such aptamers will detect new molecular fingerprints on the target cell unique to a respective disease, leading the way to biomarker discovery [44]. It was also hypothesized that the newly discovered biomarker would likely correlate with expression patterns of the same proteins in patient primary cell samples obtained in a clinical setting with the same type of disease [45]. One of the aptamers termed Sgc8 was later utilized as a capturing molecule to identify the target protein [46]. Moreover, by introducing the concept of counter-selection utilizing B-cell line Burkitt’s lymphoma to eliminate the accumulation of sequences biased towards membrane proteins, Shangguan et al. and coworkers demonstrated that specific aptamers toward a single cell type could be identified toward a specific cell line. This approach also addressed the issues of non-specific sequences and lack of homology among families, both of which were encountered in the initial two studies described above.

**Figure 2:**
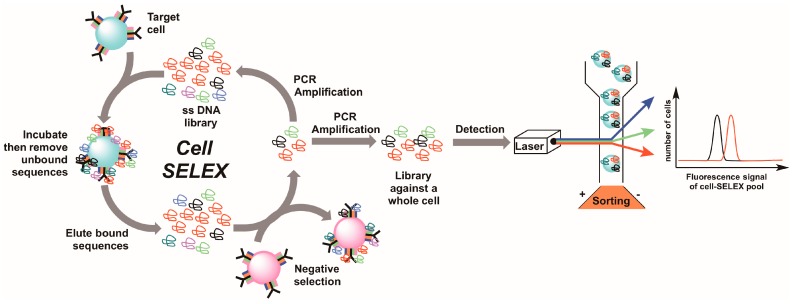
Outline of cell-SELEX with negative screening to increase selection pressure. The ability to detect enrichment of the SELEX pool using flow cytometry without altering the conditions used in the selection step enhanced the relevance of aptamers as diagnostic agents for hematopoietic diseases.

The introduction of live cell–SELEX simplified the SELEX method, allowing more laboratories to implement SELEX technology as a method to generate aptamer-based molecular probes against cells. The most important contribution of live cell–SELEX, however, is deeply rooted in the ability to detect enrichment of the pool using flow cytometry without altering the conditions used in the selection step (Figure 2). While aptamers are continuously compared to antibodies, an early hallmark of antibody success is associated with their use as effective diagnostic tools, not as therapeutics. This is especially true for the diagnosis of hematopoietic diseases [47,48,49]. Similarly, the use of flow cytometry in detecting enrichment and aptamer binding advanced the relevance of aptamers as robust diagnostic tools. Also, the elimination of radioactive ^32^P labeling of pools to monitor the progress of selection expanded these technologies to laboratories that previously lacked the specialized equipment.

Next, aptamers selected using live cell–SELEX also showed specific binding when leukemia cells were mixed with cells obtained from human peripheral blood mononuclear cell (PBMC) samples. For the first time, Shangguan et al. further demonstrated the clinical utility of aptamers in detecting primary leukemia samples [45]. In particular, using live cell-SELEX, protein tyrosine kinase 7 (PTK7) was identified as the target of candidate aptamer Sgc8 [46]. In a follow-up study in which counter-selection was eliminated, aptamer pools were enriched against Burkitt’s lymphoma cells, demonstrating that aptamer pools could be enriched against whole cells to discover a panel of aptamers against the dominant cell-surface targets [50]. Aptamer TD05, selected against Burkitt’s lymphoma cells, was further modified as a capturing probe in a pull-down assay to discover cell-surface IgM as the aptamer’s target [51]. Both of these initial examples further confirmed that aptamers selected against whole cells could be used to profile cell lines for the differential expression of cell-surface molecules, and related primary samples could also be detected utilizing flow cytometry. This is significant because this technology is a state-of-the-art detection method for immunophenotyping using antibodies in a clinical setting, and many laboratories have now used this approach to identify aptamers against cell-surface targets [52,53,54,55,56].

More recently, live cell-SELEX has been implemented for the selection of aptamers against whole cells, and using a glioblastoma cell line, a panel of RNA aptamers was generated using another variant of live cell-SELEX termed differential SELEX [57]. Here, the idea is to introduce the negative selection step before the positive selection. Another extension of live cell-SELEX is the use of an advanced form of flow cytometry called fluorescence-activated cell sorting (FACS) [58]. Usually dead cells can absorb single-stranded nucleic acid molecules, a process which greatly suppresses selection efficiency. To circumvent these issues, FACS was utilized to discriminate live from dead cells at positive selection, thus eliminating the dead cell population. This shows the effective enrichment of a SELEX library against whole cells, as well as the enhanced efficiency of SELEX through sorting out the population of dead cells. This technique also incorporates both positive and negative selection in one round to sort out the negative and positive cells, leading to a more efficient enrichment.

Since the introduction of live cell-SELEX, studies reporting on the technology have increased exponentially, and many aptamers have been identified utilizing this method, including aptamers with internalization capacity [59]. In addition, using an expanded genetic code, artificial nucleic acids were introduced to select aptamers containing two expanded nucleotides not naturally available. This recent SELEX innovation, termed Artificially Expanded Genetic Information System (AEGIS), is a novel platform showing that aptamers with modified nucleic acids can also be utilized [60]. However, of the hundreds of aptamers generated thus far from live cell-SELEX, only a few targets have been isolated and identified. Target identification of aptamers generated from live cell-SELEX is commonly performed by proteomics analysis of aptamer affinity pull-down targets. Since cell-surface membrane proteins have low solubility, these pull-down assays have, in many cases, failed [61]. Also, the amount of protein obtained in each pull-down experiment utilizing cell-surface molecules with native expression levels is often very low, complicating mass spectrometry identification [61]. Thus, new variants of live cell-SELEX have been introduced to circumvent some of the issues of live cell-SELEX with tailored approaches based on the end goal. This strategy is highly desirable, especially when aptamers are intended for use in drug delivery.

## 6. Cell-Internalizing SELEX

**Figure 3:**
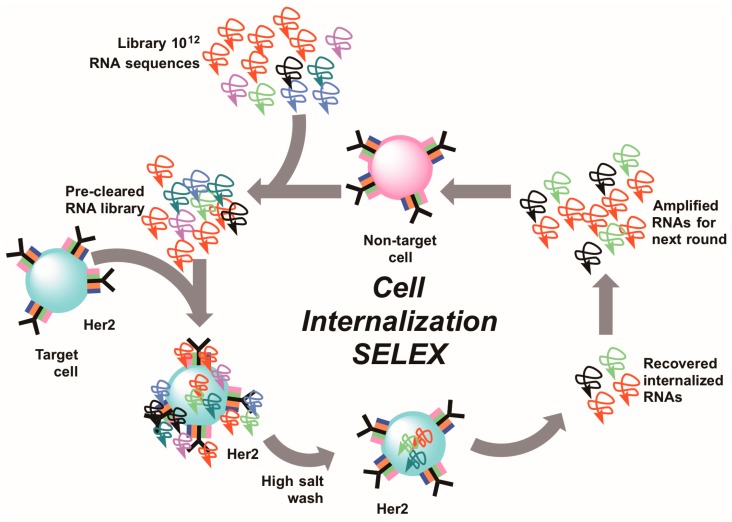
Schematic of cell-internalization SELEX. Cell-internalization SELEX was designed to isolate ssRNA sequences exclusively based on their capacity to bind the cell-surface target and their ability to rapidly internalize the cell in physiological conditions, while effectively eliminating aptamers that lack internalizing ability and aptamers that only slowly internalize into the cells.

By modifying live cell-SELEX to allow the isolation of aptamers with internalization capability, cell-internalizing SELEX was introduced [62]. One of the key features of this method is the ability to isolate ssRNA sequences exclusively based on their capacity to bind the cell-surface target and their ability to rapidly internalize the cell in physiological conditions (Figure 3). Thus, this approach effectively eliminates aptamers that lack internalizing ability and aptamers that only slowly internalize into the cells, while favoring those that rapidly internalize as they bind their cognate receptor targets. Using a derived cell line over-expressing Her2, a transmembrane receptor over-expressed in breast cancer, as the positive cell line, and a cell line not expressing Her2, Theil et al. identified a panel of aptamers against Her2 [62]. This method also utilized a stringent salt wash step to rule out the possibility of aptamers remaining on the surface. Interestingly, three aptamers identified in this study were shown to have affinity towards recombinant purified Her2. 

Although cell-internalizing SELEX demonstrated that aptamers could be isolated based on their internalization ability, it is possible that some of those aptamers will be lost due to the complexity of the method itself. The requirement of engineering a cell line with the desired over-expressed receptor and a cell line without the same receptor for the negative control makes this approach laborious and limited. These issues were addressed in a recently reported variant of cell-internalizing SELEX described by Laboni et al. [63]. Using nuclease-resistant 2′-fluoro-pyrimidines, an aptamer library was first used for differential cell-SELEX to enrich the aptamer population toward the glioma-specific cell line U87MG. After 13 rounds of differential cell–SELEX, aptamers with internalizing capacity were isolated. The target of the dominant aptamer, GL56, as identified utilizing pull-down assay, appeared to bind to the insulin receptor.

While these methods introduced alternative avenues to select aptamers based on the goal of target identification, each approach has its own limitations. For example, cell-internalizing SELEX requires the generation of two cloned cell lines, one that over-expresses the target and one that does not. Moreover, the internalization of aptamers and isolation of aptamers, once they have been internalized, is a complex and laborious process. Hybrid-SELEX requires over-expression and purification of surface proteins, and it is possible that aptamers bound to multiple receptors at the “cross-over stage” will be lost. Finally, aptamers generated using live cell–SELEX need post-SELEX manipulation of the whole cell to identify the target protein. Difficulties associated in identification of target proteins of the aptamers identified using live cell–SELEX have limited the potential application of these aptamers in developing therapeutics or diagnostics.

## 7. Ligand-Guided Selection (LIGS)

**Figure 4:**
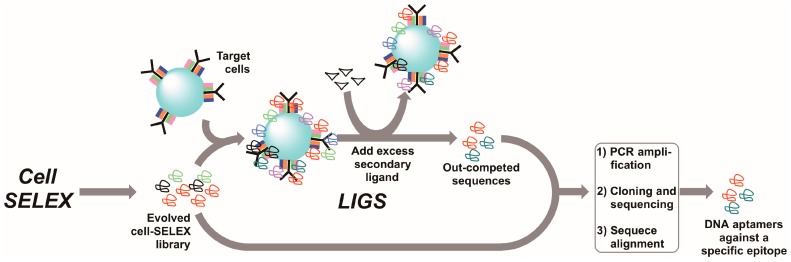
Schematic of ligand-guided selection. This approach effectively utilizes secondary ligands to selectively elute specific aptamers against a known surface marker.

When selecting aptamers against surface markers, it is important to use a target closer to its native conformation [64,65]. Recently, another variant of live cell-SELEX, termed ligand-guided selection, or LIGS, was introduced to identify aptamers against known surface proteins (Figure 4). Two initial reports focused on selecting aptamers against two dominant markers on lymphoma and leukemia cells. This method addresses some of the challenges associated with established SELEX methods against cell-surface proteins. For example, LIGS identifies highly selective aptamers against a predetermined epitope expressed on the cell surface at its native expression levels. Interrupting the selection and enrichment process, LIGS introduces a strong, highly bivalent binder, such as a monoclonal antibody (mAb), which interacts with its cognate epitope to outcompete and replace specific aptamers from an enriched SELEX pool.

Thus, the first advantage lies in a ligand having the capacity to initiate a conformational switch resulting in destabilization of the aptamer-protein complex, in turn allowing the elution of specific aptamers. For example, most growth factor receptors respond to the binding of growth factor, which triggers the conformational switch, but small molecules and ions can also trigger the conformational switch on cell-surface markers. This conformational switching of the target protein can effectively be utilized as an avenue to selectively elute specific aptamers. The second advantage lies in the combinatorial nature of the SELEX libraries. For example, at any given concentration of a SELEX pool, the individual aptamer concentration towards a specific cell-surface target is substantially low. Thus, the introduction of the pre-existing ligand above its dissociation constant towards the same surface target most likely out-competes the aptamers from this pool. Combining these two key advantages, LIGS allows the selection of aptamers against a surface marker without requiring pre- or post-SELEX sample manipulations to identify aptamer targets. The simplicity of selective elution could also be utilized to identify aptamers using primary cell samples. Utilizing LIGS, aptamers were identified against membrane IgM expressed on B-cell lymphoma and CD3 expressed in T cell leukemia. Selective elution using a secondary ligand against the same target expands the capabilities of live cell-SELEX. One of the challenges of LIGS, however, is its limited use in biomarker discovery since the method is confined to selecting aptamers from known surface markers with known high-affinity secondary ligands towards the same ligand.

## 8. Conclusions

Two key avenues characterize the most recent progress in the development of NAAs, one being the introduction of modified nucleic acids to enhance the structural diversity and, hence, the affinity of selected aptamers, and the other being the introduction of SELEX methods tailored towards a specific application to select aptamers [66]. Significant progress has been made in developing modified NAs. For example, Slow Off-rate Modified Aptamer (SOMAmer) protein-binding reagents combine the properties of the structural diversity of amino acids to generate high-affinity modified nucleic acid aptamers [67]. Next-generation aptamers, or X-aptamers (XAs), have been developed by either random or direct modification, particularly of the 5-position of certain uridines on a monothophosphate backbone of an aptamer. The approach uses chemical linkers that allow additional modification of nucleic acid bases with “X-ligands”, to enhance the structural diversity [68]. Next, the method utilizes the X-aptamer library conjugated to polystyrene beads, generating one-bead-one-sequence, to select X-aptamers with high affinity against a number of targets. In another example, click chemistry was used to attach azide sugars to an unnatural alkyne-containing uridine derivative, followed by successful incorporation into SELEX from which glycosylated DNA aptamers were evolved. Using this method, aptamers binding to the neutralizing HIV antibody 2G12 were identified [69,70]. Utilizing a similar but extended approach termed click-SELEX, aptamers against Cycle 3-GFP (Green Fluorescence Protein) were generated [71]. These examples show the potential application of modified aptamers against cell-surface targets with the aim of developing therapeutic or diagnostic tools. The enhancement of structural diversity is important in strengthening affinity and, hence, specificity towards the target. However, despite success in diversifying the library, the identification of the specific aptamers against respective targets heavily relies on the screening technology. Therefore, it is essential to improve both the screening technology of SELEX and the diversity of SELEX libraries in order to address current challenges and push the field of aptamer development forward.

With each milestone in the advancement of SELEX technology, this review summarizes how the NAA field is rapidly evolving. In particular, significant progress has been made in pushing aptamer selection technology toward generating aptamers against targets in their native state. Progress has also been made in sequencing technologies, such as next-generation sequencing (NGS), and engineered polymerases are utilized in PCR. Application of such improved technologies enables researchers in the field to explore the potential of SELEX and the combinatorial library, which is the basis of selection. Implementing flow cytometry has made it possible to detect the enrichment of SELEX pools using cells under conditions identical to those at the in vitro step, allowing the generation of high-affinity aptamers. Finally, the recently introduced LIGS technology allows the identification of aptamers against a known surface target utilizing a competing secondary ligand against the same target. With all these improvements, the future of NAA research will see considerable momentum toward translational applications.

## References

[1] FitzGerald G.A. (2016). Measure for measure: Biomarker standards and transparency. Sci. Transl. Med..

[2] Strimbu K., Tavel J.A. (2010). What are biomarkers?. Curr. Opin. HIV AIDS.

[3] Qin C., Tao L., Phang Y.H., Zhang C., Chen S.Y., Zhang P., Tan Y., Jiang Y.Y., Chen Y.Z. (2015). The assessment of the readiness of molecular biomarker-based mobile health technologies for healthcare applications. Sci. Rep..

[4] Dickmann L.J., Ware J.A. (2016). Pharmacogenomics in the age of personalized medicine. Drug Discov. Today Technol..

[5] Jain K.K. (2010). The Handbook of Biomarkers.

[6] Hassan E.M., Willmore W.G., DeRosa M.C. (2016). Aptamers: Promising tools for the detection of circulating tumor cells. Nucleic Acid Ther..

[7] Keefe A.D., Pai S., Ellington A. (2010). Aptamers as therapeutics. Nat. Rev. Drug Discov..

[8] Jayasena S.D. (1999). Aptamers: An emerging class of molecules that rival antibodies in diagnostics. Clin. Chem..

[9] Eaton B.E., Gold L., Zichi D.A. (1995). Let’s get specific: The relationship between specificity and affinity. Chem. Biol..

[10] White R.R., Sullenger B.A., Rusconi C.P. (2000). Developing aptamers into therapeutics. J. Clin. Investig..

[11] Ireson C.R., Kelland L.R. (2006). Discovery and development of anticancer aptamers. Mol. Cancer Ther..

[12] Schmidt K.S., Borkowski S., Kurreck J., Stephens A.W., Bald R., Hecht M., Friebe M., Dinkelborg L., Erdmann V.A. (2004). Application of locked nucleic acids to improve aptamer in vivo stability and targeting function. Nucleic Acids Res..

[13] Healy J.M., Lewis S.D., Kurz M., Boomer R.M., Thompson K.M., Wilson C., McCauley T.G. (2004). Pharmacokinetics and biodistribution of novel aptamer compositions. Pharm. Res..

[14] Watson S.R., Chang Y.F., O’Connell D., Weigand L., Ringquist S., Parma D.H. (2000). Anti-l-selectin aptamers: Binding characteristics, pharmacokinetic parameters, and activity against an intravascular target in vivo. Antisense Nucleic Acid Drug Dev..

[15] Lyu Y., Chen G., Shangguan D., Zhang L., Wan S., Wu Y., Zhang H., Duan L., Liu C., You M. (2016). Generating cell targeting aptamers for nanotheranostics using cell-selex. Theranostics.

[16] Maier K.E., Levy M. (2016). From selection hits to clinical leads: Progress in aptamer discovery. Mol. Ther. Methods Clin. Dev..

[17] Drolet D.W., Green L.S., Gold L., Janjic N. (2016). Fit for the eye: Aptamers in ocular disorders. Nucleic Acid Ther..

[18] Woodruff R.S., Sullenger B.A. (2015). Modulation of the coagulation cascade using aptamers. Arterioscler. Thromb. Vasc. Biol..

[19] McKeague M., Derosa M.C. (2012). Challenges and opportunities for small molecule aptamer development. J. Nucleic Acids.

[20] Catuogno S., Esposito C.L., de Franciscis V. (2016). Aptamer-mediated targeted delivery of therapeutics: An update. Pharmaceuticals.

[21] Esposito C.L., Catuogno S., de Franciscis V. (2014). Aptamer-mediated selective delivery of short RNA therapeutics in cancer cells. J. RNAi Gene Silenc..

[22] Rajendran M., Ellington A.D. (2003). In vitro selection of molecular beacons. Nucleic Acids Res..

[23] Gilboa-Geffen A., Hamar P., Le M.T., Wheeler L.A., Trifonova R., Petrocca F., Wittrup A., Lieberman J. (2015). Gene knockdown by epcam aptamer-siRNA chimeras suppresses epithelial breast cancers and their tumor-initiating cells. Mol. Cancer Ther..

[24] McNamara J.O., Andrechek E.R., Wang Y., Viles K.D., Rempel R.E., Gilboa E., Sullenger B.A., Giangrande P.H. (2006). Cell type-specific delivery of sirnas with aptamer-sirna chimeras. Nat. Biotechnol..

[25] Iaboni M., Russo V., Fontanella R., Roscigno G., Fiore D., Donnarumma E., Esposito C.L., Quintavalle C., Giangrande P.H., de Franciscis V. (2016). Aptamer-miRNA-212 conjugate sensitizes NSCLC cells to TRAIL. Mol. Ther. Nucleic Acids.

[26] Esposito C.L., Catuogno S., de Franciscis V. (2016). Aptamer-miRNA conjugates for cancer cell-targeted delivery. Methods Mol. Biol..

[27] Robertson D.L., Joyce G.F. (1990). Selection in vitro of an RNA enzyme that specifically cleaves single-stranded DNA. Nature.

[28] Tuerk C., Gold L. (1990). Systematic evolution of ligands by exponential enrichment: RNA ligands to bacteriophage t4 DNA polymerase. Science.

[29] Ellington A.D., Szostak J.W. (1990). In vitro selection of rna molecules that bind specific ligands. Nature.

[30] Lee J.F., Stovall G.M., Ellington A.D. (2006). Aptamer therapeutics advance. Curr. Opin. Chem. Biol..

[31] Chakravarthy U., Adamis A.P., Cunningham E.T., Goldbaum M., Guyer D.R., Katz B., Patel M., VEGF Inhibition Study in Ocular Neovascularization (V.I.S.I.O.N.) Clinical Trial Group (2006). Year 2 efficacy results of 2 randomized controlled clinical trials of pegaptanib for neovascular age-related macular degeneration. Ophthalmology.

[32] Ng E.W., Shima D.T., Calias P., Cunningham E.T., Guyer D.R., Adamis A.P. (2006). Pegaptanib, a targeted anti-VEGF aptamer for ocular vascular disease. Nat. Rev. Drug Discov..

[33] Sundaram P., Kurniawan H., Byrne M.E., Wower J. (2013). Therapeutic RNA aptamers in clinical trials. Eur. J. Pharm. Sci..

[34] Chu T.C., Twu K.Y., Ellington A.D., Levy M. (2006). Aptamer mediated siRNA delivery. Nucleic Acids Res..

[35] Dassie J.P., Liu X.Y., Thomas G.S., Whitaker R.M., Thiel K.W., Stockdale K.R., Meyerholz D.K., McCaffrey A.P., McNamara J.O., Giangrande P.H. (2009). Systemic administration of optimized aptamer-siRNA chimeras promotes regression of PSMA-expressing tumors. Nat. Biotechnol..

[36] Dassie J.P., Hernandez L.I., Thomas G.S., Long M.E., Rockey W.M., Howell C.A., Chen Y., Hernandez F.J., Liu X.Y., Wilson M.E. (2014). Targeted inhibition of prostate cancer metastases with an RNA aptamer to prostate-specific membrane antigen. Mol. Ther..

[37] Shamah S.M., Healy J.M., Cload S.T. (2008). Complex target selex. Accounts Chem. Res..

[38] Morris K.N., Jensen K.B., Julin C.M., Weil M., Gold L. (1998). High affinity ligands from in vitro selection: Complex targets. Proc. Natl. Acad. Sci. USA.

[39] Hicke B.J., Marion C., Chang Y.F., Gould T., Lynott C.K., Parma D., Schmidt P.G., Warren S. (2001). Tenascin-C aptamers are generated using tumor cells and purified protein. J. Biol. Chem..

[40] Sun H., Zhu X., Lu P.Y., Rosato R.R., Tan W., Zu Y. (2014). Oligonucleotide aptamers: New tools for targeted cancer therapy. Mol. Ther. Nucleic Acids.

[41] Wilner S.E., Wengerter B., Maier K., de Lourdes Borba Magalhaes M., Del Amo D.S., Pai S., Opazo F., Rizzoli S.O., Yan A., Levy M. (2012). An RNA alternative to human transferrin: A new tool for targeting human cells. Mol. Ther. Nucleic Acids.

[42] Daniels D.A., Chen H., Hicke B.J., Swiderek K.M., Gold L. (2003). A tenascin-C aptamer identified by tumor cell selex: Systematic evolution of ligands by exponential enrichment. Proc. Natl. Acad. Sci. USA.

[43] Shangguan D., Li Y., Tang Z., Cao Z.C., Chen H.W., Mallikaratchy P., Sefah K., Yang C.J., Tan W. (2006). Aptamers evolved from live cells as effective molecular probes for cancer study. Proc. Natl. Acad. Sci. USA.

[44] Mallikaratchy P., Zumrut H., Ara N., Tan W., Fang X. (2015). Discovery of biomarkers using aptamers evolved in cell-SELEX method. Aptamers Selected by Cell-SELEX for Theranostics.

[45] Shangguan D., Cao Z.C., Li Y., Tan W. (2007). Aptamers evolved from cultured cancer cells reveal molecular differences of cancer cells in patient samples. Clin. Chem..

[46] Shangguan D., Cao Z., Meng L., Mallikaratchy P., Sefah K., Wang H., Li Y., Tan W. (2008). Cell-specific aptamer probes for membrane protein elucidation in cancer cells. J. Proteome Res..

[47] Jennings C.D., Foon K.A. (1997). Flow cytometry: Recent advances in diagnosis and monitoring of leukemia. Cancer Investig..

[48] Bray R.A., Landay A.L. (1989). Identification and functional characterization of mononuclear cells by flow cytometry. Arch. Pathol. Lab. Med..

[49] Landay A.L., Muirhead K.A. (1989). Procedural guidelines for performing immunophenotyping by flow cytometry. Clin. Immunol. Immunopathol..

[50] Tang Z., Shangguan D., Wang K., Shi H., Sefah K., Mallikratchy P., Chen H.W., Li Y., Tan W. (2007). Selection of aptamers for molecular recognition and characterization of cancer cells. Anal. Chem..

[51] Mallikaratchy P., Tang Z., Kwame S., Meng L., Shangguan D., Tan W. (2007). Aptamer directly evolved from live cells recognizes membrane bound immunoglobin heavy mu chain in Burkitt’s lymphoma cells. Mol. Cell. Proteom..

[52] Zhang N., Bing T., Shen L., Song R., Wang L., Liu X., Liu M., Li J., Tan W., Shangguan D. (2016). Intercellular connections related to cell-cell crosstalk specifically recognized by an aptamer. Angew. Chem. Int. Ed. Engl..

[53] Li X., An Y., Jin J., Zhu Z., Hao L., Liu L., Shi Y., Fan D., Ji T., Yang C.J. (2015). Evolution of DNA aptamers through in vitro metastatic-cell-based systematic evolution of ligands by exponential enrichment for metastatic cancer recognition and imaging. Anal. Chem..

[54] Kim Y., Wu Q., Hamerlik P., Hitomi M., Sloan A.E., Barnett G.H., Weil R.J., Leahy P., Hjelmeland A.B., Rich J.N. (2013). Aptamer identification of brain tumor-initiating cells. Cancer Res..

[55] Sefah K., Bae K.M., Phillips J.A., Siemann D.W., Su Z., McClellan S., Vieweg J., Tan W. (2013). Cell-based selection provides novel molecular probes for cancer stem cells. Int. J. Cancer.

[56] Yang M., Jiang G., Li W., Qiu K., Zhang M., Carter C.M., Al-Quran S.Z., Li Y. (2014). Developing aptamer probes for acute myelogenous leukemia detection and surface protein biomarker discovery. J. Hematol. Oncol..

[57] Cerchia L., Esposito C.L., Jacobs A.H., Tavitian B., de Franciscis V. (2009). Differential SELEX in human glioma cell lines. PLoS ONE.

[58] Mayer G., Ahmed M.S., Dolf A., Endl E., Knolle P.A., Famulok M. (2010). Fluorescence-activated cell sorting for aptamer selex with cell mixtures. Nat. Protoc..

[59] Xiao Z., Shangguan D., Cao Z., Fang X., Tan W. (2008). Cell-specific internalization study of an aptamer from whole cell selection. Chemistry.

[60] Sefah K., Yang Z., Bradley K.M., Hoshika S., Jimenez E., Zhang L., Zhu G., Shanker S., Yu F., Turek D. (2014). In vitro selection with artificial expanded genetic information systems. Proc. Natl. Acad. Sci. USA.

[61] Hou Z., Meyer S., Propson N.E., Nie J., Jiang P., Stewart R., Thomson J.A. (2015). Characterization and target identification of a DNA aptamer that labels pluripotent stem cells. Cell Res..

[62] Thiel K.W., Hernandez L.I., Dassie J.P., Thiel W.H., Liu X., Stockdale K.R., Rothman A.M., Hernandez F.J., McNamara J.O., Giangrande P.H. (2012). Delivery of chemo-sensitizing siRNAs to HER^2+^-breast cancer cells using RNA aptamers. Nucleic Acids Res..

[63] Iaboni M., Fontanella R., Rienzo A., Capuozzo M., Nuzzo S., Santamaria G., Catuogno S., Condorelli G., de Franciscis V., Esposito C.L. (2016). Targeting insulin receptor with a novel internalizing aptamer. Mol. Ther. Nucleic Acids.

[64] Zumrut H.E., Ara M.N., Maio G.E., Van N.A., Batool S., Mallikaratchy P.R. (2016). Ligand-guided selection of aptamers against T-cell receptor-cluster of differentiation 3 (TCR-CD3) expressed on jurkat.E6 cells. Anal. Biochem..

[65] Zumrut H.E., Ara M.N., Fraile M., Maio G., Mallikaratchy P. (2016). Ligand-guided selection of target-specific aptamers: A screening technology for identifying specific aptamers against cell-surface proteins. Nucleic Acid Ther..

[66] Rohloff J.C., Gelinas A.D., Jarvis T.C., Ochsner U.A., Schneider D.J., Gold L., Janjic N. (2014). Nucleic acid ligands with protein-like side chains: Modified aptamers and their use as diagnostic and therapeutic agents. Mol. Ther. Nucleic Acids.

[67] Gold L., Ayers D., Bertino J., Bock C., Bock A., Brody E.N., Carter J., Dalby A.B., Eaton B.E., Fitzwater T. (2010). Aptamer-based multiplexed proteomic technology for biomarker discovery. PLoS ONE.

[68] He W., Elizondo-Riojas M.A., Li X., Lokesh G.L., Somasunderam A., Thiviyanathan V., Volk D.E., Durland R.H., Englehardt J., Cavasotto C.N. (2012). X-aptamers: A bead-based selection method for random incorporation of druglike moieties onto next-generation aptamers for enhanced binding. Biochemistry.

[69] Temme J.S., Krauss I.J. (2015). Selma: Selection with modified aptamers. Curr. Protoc. Chem. Biol..

[70] Temme J.S., MacPherson I.S., DeCourcey J.F., Krauss I.J. (2014). High temperature selma: Evolution of DNA-supported oligomannose clusters which are tightly recognized by HIV bnab 2G12. J. Am. Chem. Soc..

[71] Tolle F., Brandle G.M., Matzner D., Mayer G. (2015). A versatile approach towards nucleobase-modified aptamers. Angew. Chem. Int. Ed. Engl..

